# Case Report: Characterization of a RAC2 R68W homozygous activating mutation causing combined immune deficiency

**DOI:** 10.3389/fimmu.2026.1723142

**Published:** 2026-01-28

**Authors:** Aléhandra Desjardins, Louis Marois, Ágnes Donkó, Guilhem Cros, Marc-Antoine Bédard, Marie-Lorna Paul, Amy P. Hsu, Emma Darbinian, Géraldine Gosse, Thomas L. Leto, Hugo Chapdelaine, Chantal Massé, Isabel Fernandez, Fabien Touzot, Herawaty Sebajang, Emilia Liana Falcone

**Affiliations:** 1 Center for Immunity, Inflammation and Infectious Diseases, Montreal Clinical Research Institute (IRCM), Montreal, QC, Canada; 2 Department of Microbiology, Infectious Diseases and Immunology, Université de Montréal, Montreal, QC, Canada; 3 Division of Allergy and Immunology, Department of Medicine, CHU de Québec, Université Laval, Quebec, QC, Canada; 4 Laboratory of Clinical Immunology and Microbiology, National Institute of Allergy and Infectious Diseases (NIAID), National Institutes of Health (NIH), Bethesda, MD, United States; 5 Division of Allergy and Clinical Immunology, Department of Medicine, Centre Hospitalier de l’Université de Montréal (CHUM), Montreal, QC, Canada; 6 Division of Pediatric Immunology and Rheumatology, Department of Pediatrics, CHU Sainte-Justine, Université de Montréal, Montreal, QC, Canada; 7 Department of Surgery, Centre Hospitalier de l’Université de Montréal (CHUM), Montreal, QC, Canada; 8 Department of Medicine, Université de Montréal, Montreal, QC, Canada; 9 Division of Microbiology and Infectious Diseases, Department of Medicine, Centre Hospitalier de l’Université de Montréal (CHUM), Montreal, QC, Canada

**Keywords:** case report, combined immunodeficiency, gain-of-function mutation, inborn errors of immunity, RAC2

## Abstract

RAC2-related immunodeficiency is a rare inborn error of immunity with a broad clinical spectrum ranging from neonatal severe combined immunodeficiency to atypical combined immunodeficiency diagnosed later in life. We describe two unrelated French-Canadian patients carrying a rare, homozygous RAC2 variant (c.202C>T; p.R68W), both presenting with combined immunodeficiency. The first patient developed recurrent bacterial respiratory infections and early bronchiectasis that initially responded to immunoglobulin replacement therapy. She subsequently experienced severe, treatment-refractory cutaneous viral infections. In adulthood, she developed gynecologic and anal neoplasms associated with chronic viral disease, requiring long-term multidisciplinary management. The second patient presented in early childhood with recurrent respiratory infections, marked lymphoproliferation, and generalized lymphadenopathy. He then developed kidney dysfunction due to light-chain deposition disease. Management included immunoglobulin therapy, and ultimately hematopoietic cell transplantation (HCT), after which he achieved sustained clinical improvement. Genetic testing identified the same homozygous p.R68W substitution in both patients. Despite significantly reduced RAC2 protein expression, patient-derived cells exhibited increased effector signaling in the homozygous state, producing a phenotype that phenocopies dominant gain-of-function RAC2 variants. Functional hyperactivation was not observed in heterozygous cells, supporting a dosage-dependent mechanism. These cases expand the clinical and functional spectrum of RAC2 deficiency and have immediate implications for clinical care. Persistent viral disease with oncogenic complications, bronchiectasis, lymphoproliferation, or progressive organ involvement should prompt consideration of RAC2 testing even beyond infancy. From a diagnostic standpoint, reliance on expression alone may be misleading; incorporating targeted signaling assays is essential for accurate variant interpretation. Therapeutically, HCT can be effective in progressive disease with organ damage, while others may require long-term medical management of chronic viral complications. Recognizing this rare, homozygous p.R68W variant and its functional consequences supports a precision-diagnosis approach to RAC2-related immunodeficiency and refines surveillance and treatment strategies for affected patients.

## 1 Introduction

Ras-related C3 botulinum toxin substrate 2 (RAC2) is a hematopoietic Rho family GTPase that cycles between GDP-bound (inactive) and GTP-bound (active) states to act as a molecular switch to regulate actin cytoskeleton dynamics and innate immune effector functions, including membrane ruffling, phagocytosis, and assembly of the nicotinamide adenine dinucleotide phosphate (NADPH) oxidase complex with superoxide production (1). Germline *RAC2* variants cause a spectrum of inborn errors of immunity, and the largest series to date (54 patients from 37 families) demonstrates that clinical presentation correlates with the functional direction of RAC2 signaling (1). This spectrum includes: 1) monoallelic dominant-negative variants (classically p.D57N) associated with leukocytosis, neutropenia, lymphopenia, impaired neutrophil function/migration and recurrent bacterial infections with leukocyte adhesion deficiency-like features; 2) homozygous loss-of-function (null) variants presenting with early common variable immunodeficiency (CVID)-like disease; 3) heterozygous constitutively active (RAS-like) gain-of-function variants causing very early-onset severe combined immunodeficiency (SCID); and 4) other activating variants associated with later-onset combined immunodeficiencies with variable expressivity and prominent sinopulmonary and viral infections (1–6). We report two unrelated French-Canadian patients homozygous for *RAC2* [c.202C>T, p.R68W] (previously noted among *RAC2* disease-associated alleles (1)), a rare *RAC2* variant associated with increased activity, yet exhibiting markedly reduced protein stability, thereby broadening the clinical and functional spectrum of RAC2-related immunodeficiency.

## 2 Case presentation

### 2.1 Case 1

**Figure 1:**
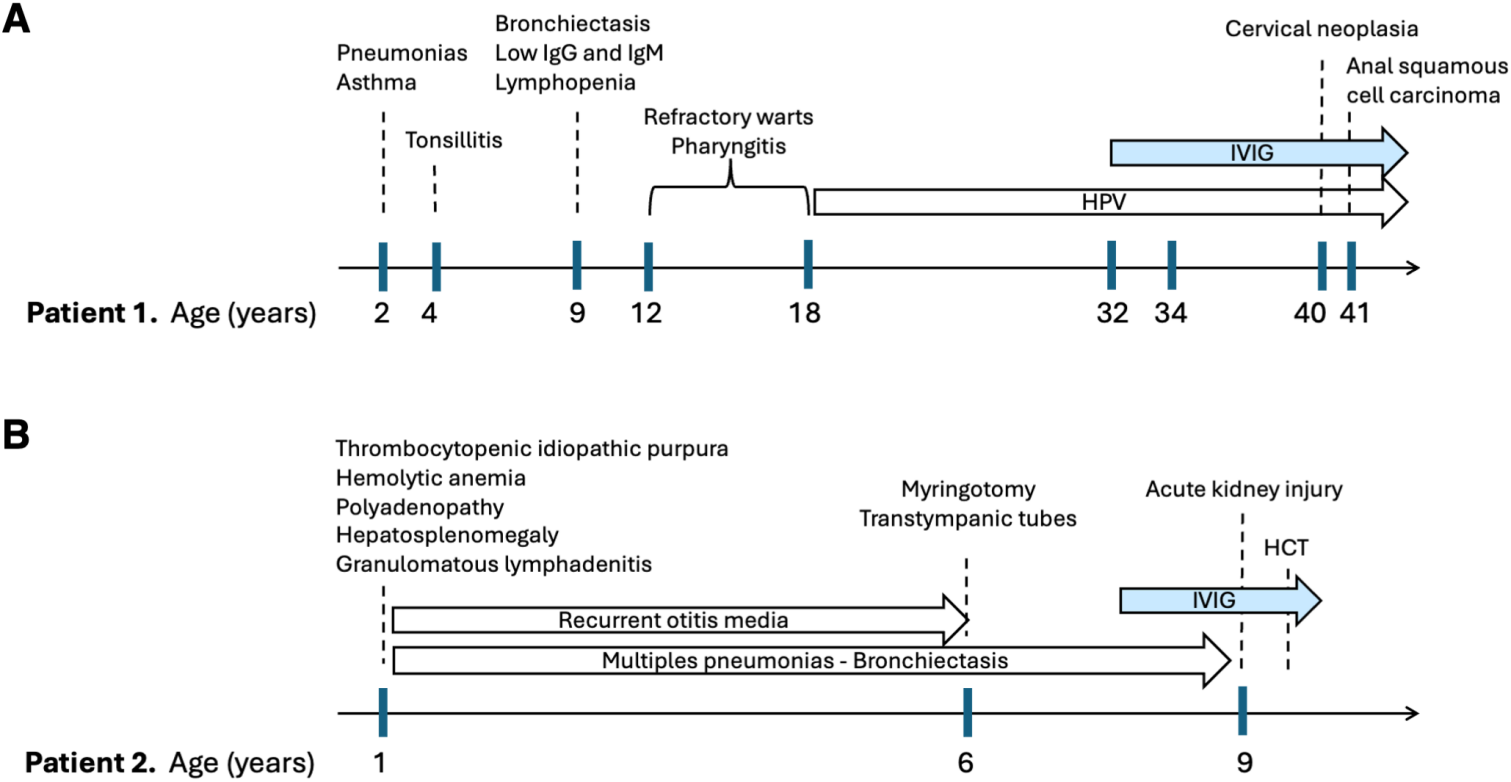
Clinical timelines of two patients with homozygous *RAC2* c.202C>T (p.R68W). Major infections, procedures, and treatments are shown chronologically for **(A)** Patient 1 and **(B)** Patient 2. Light blue arrows indicate ongoing therapies. IVIG, intravenous immunoglobulin; HCT, hematopoietic cell transplantation; HPV, human papillomavirus.

**Figure 2:**
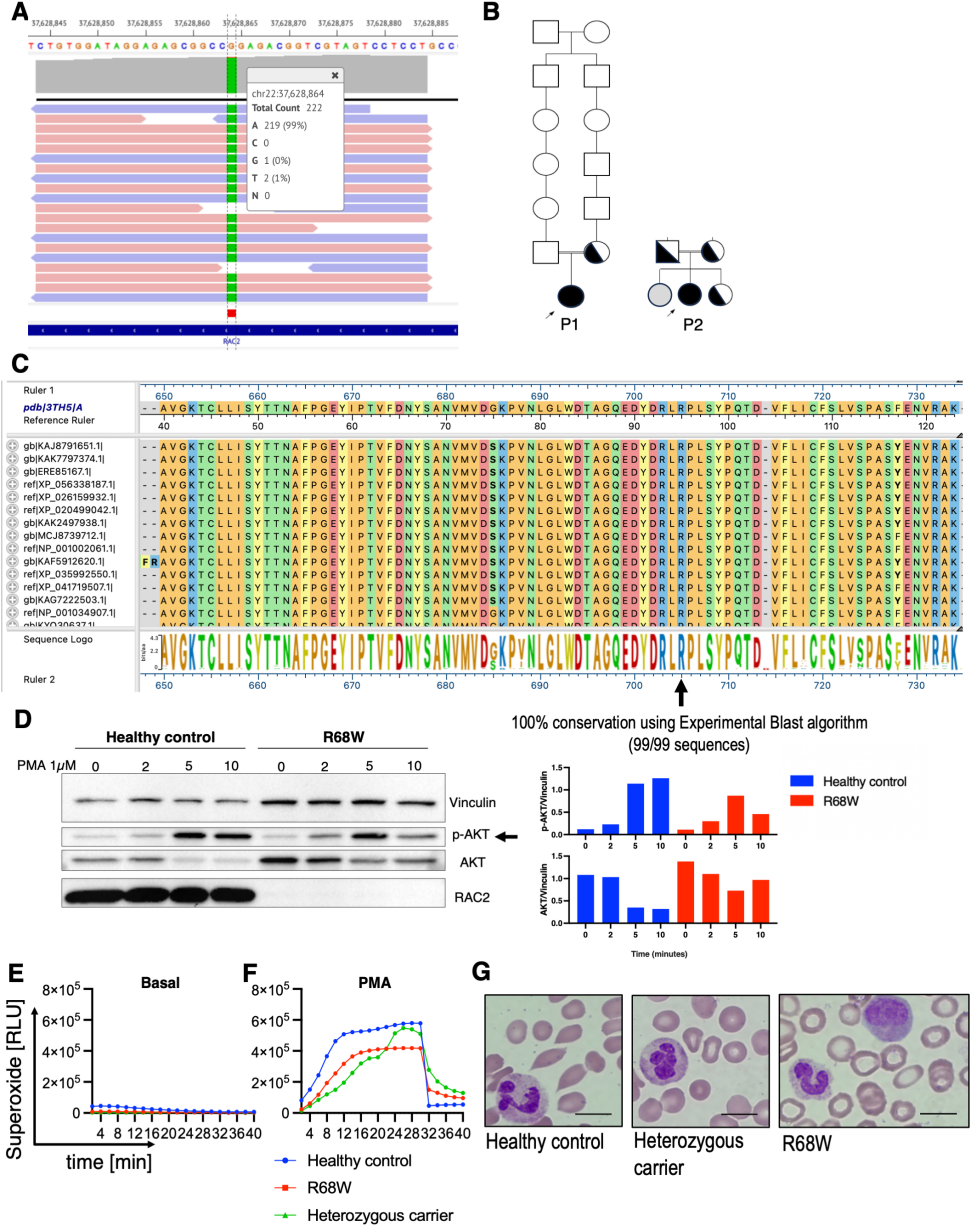
Characterization of the RAC2[R68W] variant *in vivo*. **(A)***RAC2* mutation (c.202C>T) occurs within the Switch II domain. **(B)** Family pedigrees (solid fill, homozygous; half fill, heterozygous carrier; grey fill, negative; white fill, never tested; P1: patient 1; P2: patient 2). Arrows indicate probands. **(C)***RAC2* variant sequence alignment using the BLAST Experimental Nucleotide Database and MegAlign Pro (DNAStar) showing 100% conservation across 99 representative sequences from different species. Sequence logo letter size indicates conservation (i.e., the bigger the letter, the more conserved). **(D)** Western blot and histogram show expression of RAC2, AKT and phosphorylated-AKT (p-AKT) in neutrophils from patient 1 and healthy control. Superoxide production measured by Diogenes assay and expressed relative to the healthy control under basal conditions **(E)** and after stimulation with 1µM of PMA **(F)** (n=2). **(G)** Giemsa-stained neutrophils (n=3). Scale bar = 10µm, magnification 1000X.

A 44-year-old woman was evaluated following the resection of an anal epidermoid cancer. Her past medical history was significant for recurrent pneumonias and severe asthma leading to bronchiectasis at 9 years of age, and recurrent tonsillitis resulting in tonsillectomy. As a teenager, she suffered from recurrent cold sores and persistent warts on her hands and genitals, which persisted into adulthood. Her immune tests were significant for moderate hypogammaglobulinemia and lymphopenia. She was treated with intravenous immunoglobulins for presumed common variable immunodeficiency (CVID). Worsening lung condition with increasingly frequent asthma exacerbations and numerous bronchiectasis led to lower lobe collapse which necessitated left pneumectomy. She underwent a hysterectomy at age 40 for high grade cervical and vaginal intraepithelial neoplasia, which was followed by the resection of a perianal epidermoid cancer at age 44. The timeline of events is shown in **Figure 1A**. She was found to have moderate T- and B-cell lymphopenia with reduction in naïve T lymphocytes (**Table 1**). A homozygous *RAC2* c.202C>T (p.R68W) variant was identified using a next generation sequencing (NGS) gene panel (**Figures 2A, C**). The position of pR68W within RAC2 and in relation to other disease-associated variants is shown in **Supplementary Figure S1**. The variant is rare in population databases (gnomAD v4.1 global allele frequency ~1.43×10^-5^) and is predicted to be deleterious by multiple in silico tools (CADD, MutationTaster, PolyPhen-2, and SIFT).

**Table 1:** Patient lymphocyte subset counts at time of genetic diagnosis.

Lymphocyte subsets	Patient 1				Patient 2*			
	Absolute values (x 10^6^/L)	Adult reference range (x 10^6^/L)	% of total lymphocytes	Adult reference range (%)	Absolute values (x 10^6^/L)	Pediatric reference range (x 10^6^/L)	% of total lymphocytes	Pediatric reference range (%)
Total lymphocytes	700	1450-3000	100	−	−	−	100	−
CD3+	511	943-1972	73	60-81	239	1400-3700	53	56-75
CD3+CD4+	287	525-1260	41	28-55	182	700-2200	40	28-47
CD3+CD8+	202	221-756	28.9	12-32	42	490-1300	9	16-30
CD4+CD45RA+CD31+	−	−	6	32-63	−	−	−	−
CD19+	18.9	115-540	2.7	5-26	175	390-1400	39	14-33
CD19+CD27+	−	−	7	>10	−	−	−	−
CD3-CD56+	169.4	60-414	24.2	4-19	33	130-720	7	4-17

*Patient 2 was on sirolimus at time of analysis.

The patient is from a consanguineous French-Canadian family (**Figure 2B**, left); her mother had recurrent pneumonias and her father had human papillomavirus (HPV)-mediated oropharyngeal cancer. The mother (age 66) was heterozygous for the *RAC2* variant and was the only relative available for testing. Her mother’s immunological evaluation (i.e., immunoglobulins, complement, complete blood count (CBC), lymphocyte phenotyping, vaccine responses) was normal. Autoantibody testing showed anti-double-stranded DNA (anti-dsDNA) positivity, with negative for antinuclear antibodies (ANA) and extractable nuclear antigen antibodies (ENA). She is currently only treated for emphysema.

### 2.2 Case 2

A 10-year-old girl first presented at 14 months with Evans syndrome (recurrent immune thrombocytopenia and autoimmune hemolytic anemia) refractory to prednisone and intravenous immunoglobulin. She had generalized lymphadenopathy and hepatosplenomegaly with hypermetabolic activity on PET. Treatment with rituximab and sirolimus normalized the platelet count and maintained the hemoglobin levels. Sirolimus was selected as an immunomodulatory, steroid-sparing agent for the auto-immune cytopenias and lymphoproliferative disease. A para-aortic lymph-node biopsy showed benign granulomatous lymphadenitis. She had recurrent acute otitis media, requiring myringotomy with tympanostomy tubes at age 6, as well as multiple pneumonias leading to bronchiectasis. Childhood asthma was diagnosed after cystic fibrosis and primary ciliary dyskinesia were excluded. She also developed extensive molluscum contagiosum.

From age 1, immunologic evaluation showed failure to mount protective antibody responses to pneumococcal, *Haemophilus influenzae* type b, tetanus, and diphtheria vaccines. Laboratory studies demonstrated hypergammaglobulinemia (IgG up to 45.9 g/L; reference 6.5–16.0 g/L) with monoclonal gammopathy (IgG M-spike initially 20.2 g/L, rising to 26 g/L by age 6) (**Table 1**). At age 7, she developed acute kidney injury attributed to IgG immune-complex deposition with kappa light-chain deposition disease, treated with daratumumab, bortezomib, and dexamethasone.

A targeted NGS panel identified a homozygous missense variant in *RAC2* (c.202C>T, p.R68W) and a homozygous nonsense variant in *TNFRSF9* (c.742G>T, p.Q248*). She underwent an unrelated hematopoietic cell transplant at age 9, with a favorable post-transplant course. The clinical timeline is shown in **Figure 1B**.

The patient is from a consanguineous French-Canadian family (**Figure 2B**, right). Both parents are first- cousins, are clinically healthy, and are heterozygous carriers of *RAC2* and *TNFRSF9* variants. The patient’s sister is healthy and asymptomatic; she is homozygous for the *TNFRSF9* variant but heterozygous for the *RAC2* variant, and her immune evaluation is normal.

## 3 Functional characterization of the RAC2[R68W] variant

Neutrophils were isolated from peripheral blood, and protein expression (RAC2, AKT, phospho-AKT (p-AKT), and vinculin as loading control) was assessed by Western blot. Superoxide production was quantified in unstimulated and phorbol 12-myristate 13-acetate (PMA)-stimulated neutrophils using the Diogenes chemiluminescent assay (1, 2). For functional studies, HEK293 and COS-7 cells were transiently transfected with plasmids encoding wild-type (WT) RAC2 or RAC2 p.R68W together with NADPH oxidase components (gp91^phox^/NOX2, p67^phox^, p47^phox^) and GFP (2), using Lipofectamine LTX Plus according to the manufacturer’s instructions. Superoxide generation and protein expression were measured using Diogenes assays and Western blotting, respectively. Active RAC2 pull-down was performed in RAC2-transfected COS-7 cells using purified PAK1 p21-binding domain (PBD)-GST fusion protein (7) coupled to glutathione-agarose beads. Bound proteins were eluted and probed by Western blot (1, 2).

RAC2 was undetectable in neutrophils from Patient 1 by immunoblot. Upon PMA stimulation, p-AKT increased from baseline but remained slightly lower than in the healthy control (**Figure 2D**). Despite this, PMA-induced superoxide generation was comparable to that of the heterozygous carrier and the healthy control (**Figures 2E, F**). Neutrophil morphology was similar across all three individuals (**Figure 2G**).

**Figure 3:**
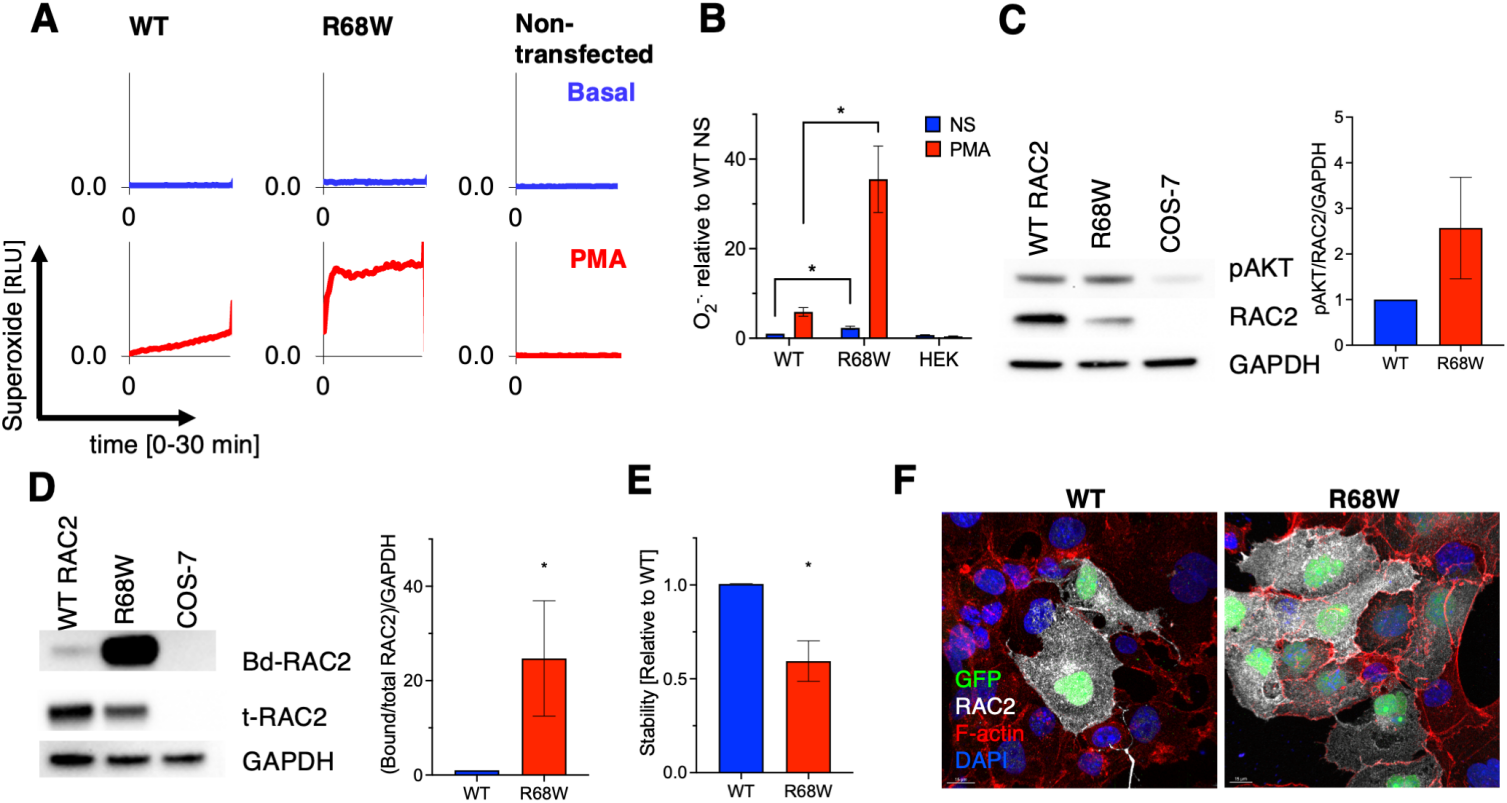
Functional characterization of the RAC2[R68W] variant *in vitro*. **(A)** Superoxide (O2-) production by HEK293 cells transfected with NADPH oxidase components and RAC2 (wild-type [WT] RAC2, RAC2[R68W] variant or non-transfected) under basal or PMA-stimulated conditions. **(B)** Integrated kinetics of basal (NS) and PMA-stimulated conditions normalized to WT RAC2 non-stimulated (n=3). **(C)** Western blot of phosphorylated AKT (pAKT) of WT, RAC2[R68W] or non-transfected COS-7 cells (n=3). **(D)** PAK1-PBD binding assay, designed to pull down only the active form of RAC2, for WT, RAC2[R68W] transfected or non-transfected COS-7 cells (n=3). T-RAC2: total RAC2. **(E)** RAC2/GAPDH protein levels, normalized to WT RAC2. **(F)** Confocal fluorescent microscopy of COS-7 cells co-transfected with GFP and RAC2 proteins, Scale bar = 10 µm, magnification 400X. Bar graphs show mean ± SEM. Asterisks indicate statistical significance (*P* < 0.05).

To assess function *in vitro*, HEK293 and COS-7 cells were co-transfected with NADPH-oxidase components and either wild-type (WT) RAC2 or RAC2 p.R68W (1, 2). Upon PMA stimulation, HEK293 cells expressing p.R68W generated significantly more superoxide than WT (**Figures 3A, B**). In COS-7 cells, p-AKT was increased despite reduced RAC2 abundance (**Figure 3C**). PAK1-PBD pull-down showed a higher fraction of GTP-bound RAC2 (Bd-RAC2/total RAC2) with RAC2 p.R68W than with WT (**Figure 3D**), even though p.R68W displayed decreased protein stability (**Figure 3E**). These findings indicate constitutive hyperactivation of RAC2 signaling by RAC2 p.R68W. COS-7 cells expressing p.R68W had a slightly granular appearance but lacked the macropinosomes reported for other activating *RAC2* variants (**Figure 3F**) (1).

## 4 Discussion

We report two unrelated patients homozygous for RAC2 c.202C>T (p.Arg68Trp), a variant that yields an unstable yet hyperactive RAC2 protein. Arg68 is highly conserved across species (**Figure 1C**), consistent with functional constraint at this position. Although RAC2 p.R68W markedly reduces total RAC2 levels, an increased fraction of GTP-bound/active RAC2 likely compensates, thereby helping to preserve AKT phosphorylation and superoxide production. Thus, despite reduced total RAC2 abundance, the increased proportion of GTP-bound RAC2 together with enhanced downstream signaling supports increased specific activity rather than a null (loss-of-function) effect. Accordingly, low steady-state RAC2 detection in neutrophils by immunoblot should not be interpreted in isolation when assessing mechanism for RAC2 variants.

Similar to other activating RAC2 variants, patients with RAC2 p.R68W present with a combined immunodeficiency-like phenotype, characterized by reduced lymphocyte counts and hypogammaglobulinemia with recurrent sinopulmonary infections leading to bronchiectasis. Although formal lymphocyte proliferation/activation assays were not available to directly assess antigen-specific T-cell function, the clinical course, including susceptibility to viral infections (e.g., herpes simplex virus and HPV) and neoplasms, supports impaired cellular immune responses (1). Like p.E62K, p.R68W lies within the Switch II region of RAC2 (**Supplementary Figure S1**), a domain that regulates GTP hydrolysis and effector interactions, helping to stabilize the active (GTP-bound) state and thereby contributing to the combined immunodeficiency phenotype (3). Both RAC2 p.R68W and RAC2 p.E62K are associated with enhanced AKT signaling (2, 4). A patient with RAC2 p.N92T showed a similar clinical picture and excess neutrophil-derived superoxide (5).

The coexistence of homozygous variants in *TNFRSF9* and *RAC2* in Patient 2 complicates attribution of the phenotype. The previously unreported *TNFRSF9* c.742G>T (p.Q248*) variant introduces a premature stop codon predicted to truncate the cytoplasmic tail by eight amino acids. Reported *TNFRSF9* (CD137) deficiency has been associated with sinopulmonary infections, autoimmunity (including autoimmune hemolytic anemia), generalized lymphadenopathy, hepatosplenomegaly, and early Epstein-Barr virus (EBV)-driven lymphoma (8, 9). Notably, the patient’s sister, homozygous for *TNFRSF9* c.742G>T (p.Q248*) but heterozygous for the *RAC2* variant, is healthy with a normal immune evaluation, suggesting reduced penetrance or a milder/hypomorphic effect of this *TNFRSF9* variant and implicating *RAC2* c.202C>T (p.R68W) as the primary driver of the combined immunodeficiency in Patient 2.

These two unrelated patients homozygous for the same *RAC2* c.202C>T (p.R68W) variant demonstrate variability in clinical expression, ranging from chronic viral disease with oncogenic complications to lymphoproliferation with organ involvement. Our functional data show that p.R68W produces low steady-state protein levels yet disproportionate signaling output, consistent with constitutive hyperactivation. These observations support enhanced surveillance for chronic viral infection and malignancy and indicate that hematopoietic cell transplantation (HCT) can be effective in progressive, organ-threatening disease, whereas others may be managed medically. In published RAC2-related disease, allogeneic HCT has been used as definitive therapy in severe cases across the RAC2 functional spectrum (including dominant-negative and activating/constitutively active phenotypes), particularly when patients develop progressive, organ threatening complications or uncontrolled infections/immune dysregulation (1, 6). General guidance for HCT in inborn errors of immunity (IEI) emphasizes improved outcomes when transplantation is performed before irreversible organ damage and in experienced centers (10). In Patient 2, the development of progressive organ involvement (e.g., renal light-chain deposition disease) and severe immune dysregulation supported proceeding to HCT, with sustained clinical improvement post-transplant.

Limitations include the small sample size, the absence of formal lymphocyte proliferation/activation assays, and potential confounding from the co-inherited TNFRSF9 variant, which limit generalizability. Nonetheless, taken together, the clinical, genetic, and functional evidence indicates that homozygous *RAC2* p.R68W variants can cause a combined immunodeficiency that warrants ongoing monitoring and tailored management (e.g., IVIG, antibiotic prophylaxis, and consideration of HCT in certain cases).

## 5 Conclusion

We identified a rare homozygous *RAC2* c.202C>T (p.Arg68Trp) variant in two unrelated French-Canadian patients. The variant yields a markedly unstable yet hyperactive RAC2 protein and a clinical picture that phenocopies dominant gain-of-function RAC2 variants. These findings emphasize that evaluation of suspected RAC2-related immunodeficiency should integrate both expression and functional signaling assays, as reduced steady-state expression alone may otherwise be misinterpreted as a null (loss-of-function) effect. Recognition of this mechanism has direct management implications, including enhanced viral/malignancy surveillance and treatment ranging from IVIG and antimicrobial prophylaxis to HCT in progressive disease.

## Data Availability

The original contributions presented in the study are included in the article and **Supplementary Material**. Additional inquiries can be directed to the corresponding author.
