# Supplementary material for: Case Report: Characterization of a RAC2 R68W homozygous activating mutation causing combined immune deficiency
Source: Front Immunol. 2026 Jan 28;17:1723142. doi: 10.3389/fimmu.2026.1723142 (PMC12891230; doi:10.3389/fimmu.2026.1723142)
Supplement: Supplementary file 1 [file Image1.pdf]

Supplementary Figure S1

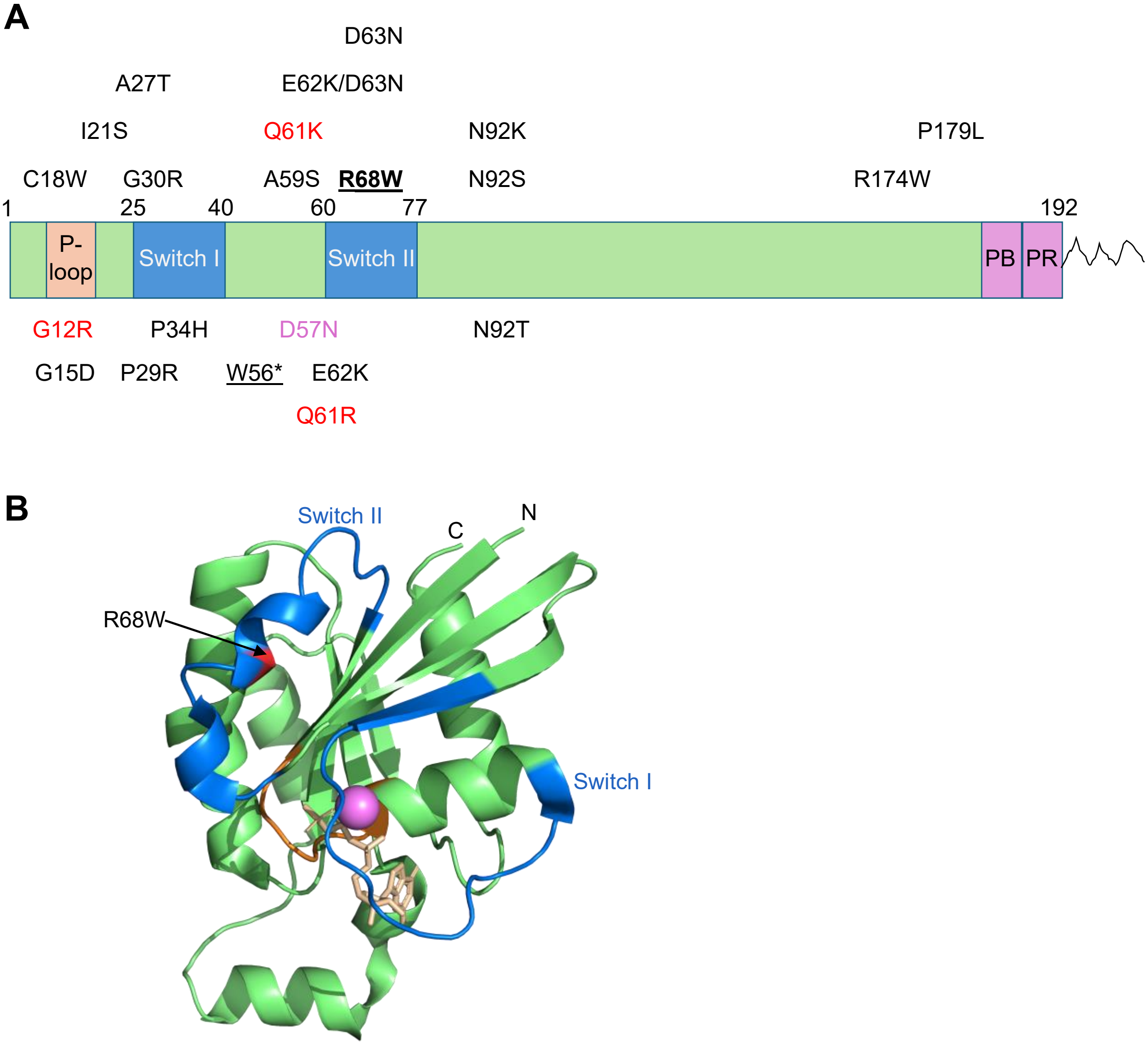

Supplementary Figure S1. Location and structural context of RAC2 disease-associated variants. (A) Schematic of RAC2 protein domains (P-loop, Switch I, Switch II, polybasic region [PB], and prenylation motif/region [PR]) with disease-associated variants mapped to their approximate positions. p.R68W (bold) denotes the variant described in this case report. Font color indicates the predominant clinical phenotype reported for each variant (red, severe combined immunodeficiency [SCID]; lavender, leukocyte adhesion deficiency [LAD]-like; black, combined immunodeficiency [CID]), and underlining indicates variants observed in the homozygous state. An asterisk (*) denotes a stop-gain (nonsense) variant. (B) Ribbon representation of RAC1 (amino acids 2-177; Protein Data Bank (PDB) ID: 3TH5) shown as a structural proxy for RAC2, with Switch regions in blue and the P-loop in orange. Mg^{2+} is shown in lavender and the non-hydrolysable GTP analog (GNP) in tan. The position corresponding to RAC2 p.R68W is highlighted in red (arrow). Structure source: Krauthammer et al., *Nat Genet* (2012) (PMID: 22842228).
